# Photobiomodulation in 3D tissue engineering

**DOI:** 10.1117/1.JBO.27.9.090901

**Published:** 2022-09-14

**Authors:** Polina Bikmulina, Nastasia Kosheleva, Anastasia Shpichka, Vladimir Yusupov, Vladimir Gogvadze, Yury Rochev, Peter Timashev

**Affiliations:** a Sechenov First Moscow State Medical University, World-Class Research Center “Digital Biodesign and Personalized Healthcare”, Moscow, Russia; b Sechenov First Moscow State Medical University, Institute for Regenerative Medicine, Moscow, Russia; c FSBSI Institute of General Pathology and Pathophysiology, Moscow, Russia; d Sechenov University, Laboratory of Clinical Smart Nanotechnologies, Moscow, Russia; e Institute of Photon Technologies of FSRC “Crystallography and Photonics” RAS, Troitsk, Russia; f Lomonosov Moscow State University, Faculty of Medicine, Moscow, Russia; g Karolinska Institutet, Institute of Environmental Medicine, Division of Toxicology, Stockholm, Sweden; h National University of Ireland, Galway, Galway, Ireland

**Keywords:** photobiomodulation, tissue engineering, regenerative medicine, low-level light

## Abstract

**Significance:**

The method of photobiomodulation (PBM) has been used in medicine for a long time to promote anti-inflammation and pain-resolving processes in different organs and tissues. PBM triggers numerous cellular pathways including stimulation of the mitochondrial respiratory chain, alteration of the cytoskeleton, cell death prevention, increasing proliferative activity, and directing cell differentiation. The most effective wavelengths for PBM are found within the optical window (750 to 1100 nm), in which light can permeate tissues and other water-containing structures to depths of up to a few cm. PBM already finds its applications in the developing fields of tissue engineering and regenerative medicine. However, the diversity of three-dimensional (3D) systems, irradiation sources, and protocols intricate the PBM applications.

**Aim:**

We aim to discuss the PBM and 3D tissue engineered constructs to define the fields of interest for PBM applications in tissue engineering.

**Approach:**

First, we provide a brief overview of PBM and the timeline of its development. Then, we discuss the optical properties of 3D cultivation systems and important points of light dosimetry. Finally, we analyze the cellular pathways induced by PBM and outcomes observed in various 3D tissue-engineered constructs: hydrogels, scaffolds, spheroids, cell sheets, bioprinted structures, and organoids.

**Results:**

Our summarized results demonstrate the great potential of PBM in the stimulation of the cell survival and viability in 3D conditions. The strategies to achieve different cell physiology states with particular PBM parameters are outlined.

**Conclusions:**

PBM has already proved itself as a convenient and effective tool to prevent drastic cellular events in the stress conditions. Because of the poor viability of cells in scaffolds and the convenience of PBM devices, 3D tissue engineering is a perspective field for PBM applications.

## 1 Introduction

Photobiomodulation (PBM) is a nonthermal process that utilizes nonionizing forms of light sources, including lasers, LEDs, and broadband light in the visible (400 to 750 nm) and infrared range (750 to 1100 nm).^1^^,^^2^ The process of PBM of biological objects is usually referred to as an irradiation.^3^^–^^5^ Historically, the PBM application in clinics began earlier than any clinical trials, *in vivo* or *in vitro* tests on this method. The PBM effects themselves were first discovered in 1967 by Endre Mester while he was trying to treat an advanced melanoma in one of his patients.^6^ Intrigued by the results of laser irradiation being opposite to those expected, Mester continued his experiments on mice—and his works are the first known confirmation of the PBM effects.^7^ Nowadays, PBM is widely utilized for various clinical and therapeutic applications. PBM in the red and NIR ranges have proven itself to be beneficial for the repair of cartilage and bone defects,^8^^,^^9^ a wide range of neuronal disorders,^10^ and is also capable of resolving pain, decreasing inflammation, and accelerating healing.^11^^–^^13^ Currently, there are more than 600 registered clinical trials related to PBM, and more than half of them have been successfully completed. The trials based on the PBM include various conditions, such as postoperative wounds, chronic pain, skin diseases, and more (details are provided in Table 1). Based on this clinical research, PBM devices using both lasers and LEDs have been cleared for marketing by FDA.^14^^–^^16^

**Table 1:** Completed clinical trials on PBM.

Number	Number of patients	Condition/disease	Results
NCT02383472	53	Mild traumatic brain injury	Positive effects on verbal and visual memory, decreased reaction time, increased visual motor speed
NCT01439724	94	Oral mucositis on the background of chemotherapy	Decreased morbidity, prevention of the oral mucositis relapses
NCT02267850	29	Orthodontic treatment time	Accelerated tooth movement
NCT02181400	21	Diabetic macular oedema	Anatomical improvement of macular oedema
NCT03741062	11	Wound healing of human palatal tissue	Improved wound healing and postoperative comfort, prevention of scars, decreased consumption of analgesic pills
NCT00929773	100	Chronic pain in neck and shoulders	Increased range of motion, decreased reported degree of pain
NCT01821352	53	Obesity	Reduction of circumference of hips, waist, and upper abdomen
NCT02588599	54	Toenail onychomycosis	Increased extent of clear nail
NCT01538199	28	Major depressive disorder	Reduced posttreatment depression ratings

The use of PBM in clinical practice was followed by gradual understanding of its mechanisms. Cytochrome С oxidase (CCO) is considered the main target of red and NIR light in a cell. PBM can influence on oxidative processes in cells, ATP production, calcium waves, and other processes associated with the mitochondria metabolism. The second messengers, such as NO, ATP, ROS, and ${\mathrm{Ca}}^{2+}$, are activated via the redox changes of the mitochondrial electron transport chain.^17^^,^^18^ This leads to the upregulation of various cellular pathways, linked to cell proliferation, differentiation, metabolic changes, antiapoptotic, or anti-inflammatory effects.^19^ With developing methods of cell biology, a deeper understanding of the PBM effects and mechanisms becomes possible. Moreover, the upcoming fields of regenerative medicine and, in particular, tissue engineering (TE), provide a new platform for the PBM application. As mentioned above, the therapeutic range of PBM wavelengths (600 to 1000 nm) is conditioned not only by the cellular susceptibility but also by the light penetration properties. The so-called optical transparency window allows PBM to permeate hydrated tissues, scaffolds, and hydrogels with high efficacy.^20^^,^^21^ In the last decades, the scope of such 3D cultivation systems’ application in the fields of biology and medicine has been constantly expanding. Scaffolds and hydrogels gradually replace classic monolayer cultures when used as drug screening platforms, native tissue models, or clinical products. 3D systems have several crucial advantages over 2D cultures, notwithstanding limitations reducing their applications. Biomaterials with required mechanical properties often can affect cell viability due to the restricted diffusion, mechanical and nutritional stress. Therefore, effective and convenient approaches to maintain the viability of 3D tissue-engineered constructs are of great interest. The PBM technique is noninvasive and does not require direct manipulations of scaffolds or cell media. Light penetrates hydrogels in the range of the most commonly used thicknesses easily. Furthermore, with the development of LED sources and semiconductor lasers (LD, laser diodes), the precise technical characterization of the applied light becomes available. Such LED and LD sources often represent mobile, compact, and controllable devices. These advantages allowed LEDs and LDs to be applied in the upcoming bioprinting approaches. Most of the existing extrusion bioprinters have ultraviolet light sources to perform the photocrosslinking of printed constructs. The practical convenience of these devices should be noted here, because bioprinting requires an accurate dose and duration of irradiation in the conditions of limited space and time. As can be seen from the diversity of both commercial and original extrusion bioprinters, LEDs and LDs match all these demands successfully. Furthermore, the supplementation of a 3D bioprinter with a red or NIR light source for PBM of cells during the printing will cause no technical issues. Several studies have already shown the potential of PBM for a better scaffold’s integration in the host tissues,^22^ promotion of vascularization,^23^ and as a preconditioning method for cell therapy.^24^^,^^25^

All these factors make it rational and effective to use red and NIR light with optical transparent scaffolds laden with weakened cells. However, due to the diversity of PBM sources and parameters, cell types, scaffold compositions, and geometries, it is hard to predict whether the chosen combination of factors would be effective or not. In this review, we aim to define the key points regarding PBM of cells in 3D scaffolds for outlining the optimal strategies of PBM application in TE. We first define the main limitations of 3D cell cultivation systems and some PBM properties, which can help to overcome these limitations. Next, we describe the optical properties of tissue-engineered constructs and available sources and parameters of PBM. Finally, we discuss the main mechanisms and cellular pathways triggered by PBM and the following outcomes of PBM of cells in scaffolds to define promising strategies for the cell survival stimulation in 3D scaffolds.

## 2 Tissue-Engineered Constructs: Promises and Limitations

To date, tissue-engineered constructs consisting of scaffolds and cells represent one of the foremost branches of regenerative medicine. 3D cultivation systems offer the *in vivo*-like conditions for cells due to the presence of extracellular matrix (ECM), cell–ECM contacts, mechanical signals, and nutritional and chemical gradients.^26^^–^^28^ These systems are applicable for native tissue modeling, disease mechanism investigation, drug screening, and cell therapy.^29^^–^^35^ However, various 3D systems often have drawbacks such as restricted diffusion and lack of vascularization, which can lead to hypoxia, nutritional stress, and cell death.^36^^,^^37^

### 2.1 Altered Diffusion of Macromolecules and Oxygen in Tissue-Engineered Constructs

Diffusion coefficients of molecules can vary depending on the diverse scaffold’s features. First of all, the molecule size, scaffold-building proteins’ concentration, and the rates of cell metabolic activity should be mentioned.^38^ The diffusion transport is also defined by the structural properties of the scaffold, such as the porosity, pore size, overall linear size, tortuosity, microcavities, and geometrical features.^39^

The average diffusion coefficients of dextran in human skin are ${9*10}^{-12}\;m^{2}\;s^{-1}$ for 500 kDa and $2.3*10^{-11}\;m^{2}\;s^{-1}$ for 40 kDa.^40^ As for macroporous scaffolds, the diffusion coefficient of small molecules (oxygen, glucose, calcium, phosphates) is typically around $10^{-9}$ to $10^{-10}\;m^{2}\;s^{-1}$, and for the larger ones (molecular weights 4.4 kDa to 2 MDa) it lies in the range of $10^{-10}$ to $10^{-11}\;m^{2}\;s^{-1}$.^41^^–^^43^ As observed in Ref. 38, the diffusion coefficient of oxygen in different biomaterials varies from 0.24 to $2.5{*10}^{-9}\;m^{2}\;s^{-1}$, whereas in water it is $2.7*10^{-9}\;m^{2}\;s^{-1}$. Some studies, however, show that the extracellular matrix does not restrict diffusion of small molecules.^44^ According to Ref. 38, the determining factor for the oxygen level inside a construct is not the polymer concentration, but the cell density. The oxygen consumption in a 3D construct is influenced by the monolayer cell culture properties before seeding onto a scaffold. Cells cultured under low confluency consume oxygen rapidly, which causes the oxygen level to drop to almost zero 8 to 10 h after the inoculation.^45^

An appropriate oxygen level is one of the crucial conditions for the normal cell physiology. Usually, cells are cultivated in normoxia (21% oxygen), although the *in vivo* oxygen level is considered to be around 5% to 8%.^46^^,^^47^ Low oxygen tension has been shown to maintain an active state of stem and progenitor cell populations.^48^ On the other hand, the lack of oxygen in a tissue-engineered construct can reduce the cell viability.^38^^,^^41^^,^^49^ Typical oxygen diffusion distances in the tissues are restricted to 100 to 150  μm.^50^ In case of exceeding this value, after a few days of cultivation, the oxygen levels inside the scaffold drop dramatically, which causes cell death.^51^ Some of the authors consider glucose levels and not oxygen the main limiting factor.^52^ Average diffusion distances for such metabolites as glucose are in the range between 5 and 200  μm.^50^

**Fig. 1:**
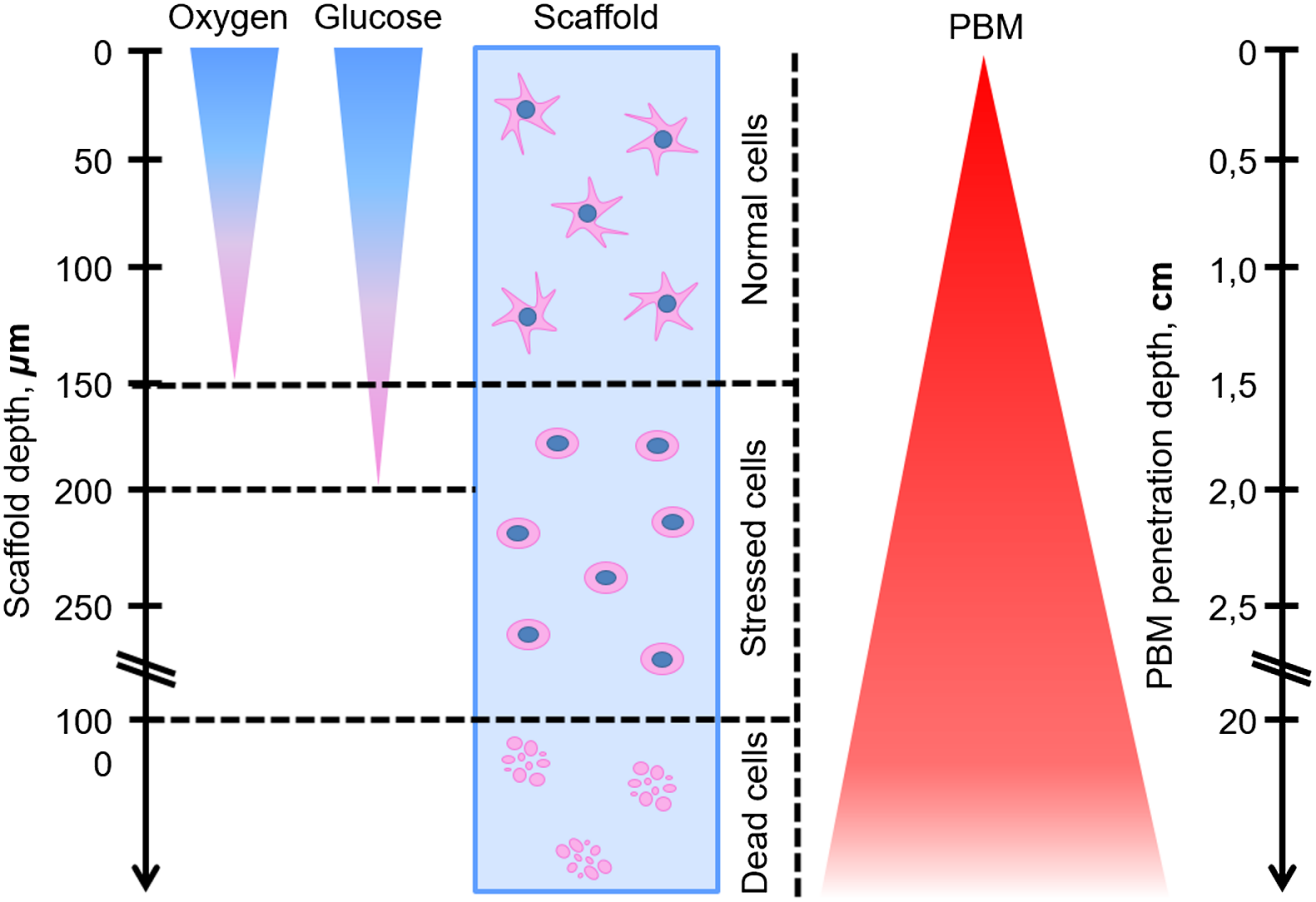
Average diffusion distances of oxygen and nutrients (glucose) matched with cell viability on different depths of the scaffold and PBM penetration abilities. The diffusion distance for oxygen is ∼150  μm, for glucose is 200  μm. In a range of 0 to 150  μm of scaffold cells are metabolically active and viable.^50^ After reaching diffusion limits, cells are exposed to deprivation of oxygen and nutrients. As a result, proliferation, metabolic activity, and general viability decrease. At depths more than 1 mm cell death occurs.^54^ Depending on tissue/scaffold and light source type, PBM penetration depth varies from 2 to 3 to 23 cm.^58^^–^^60^

The restricted diffusion is aggravated by the lack of vascularization. Cells in a construct can be distanced as far as a few millimeters from the closest capillary, whereas in native tissues these distances do not exceed 20 to 30  μm.^53^ In a static culture conditions, parts of tissue-like constructs outlying the surface more than 0.5 to 1 mm contain only dead cells.^54^ If the depth of a construct goes beyond 100 to 200  μm, the cell viability drops significantly due to the nutritional stress and oxygen deprivation^55^^–^^57^ (Fig. 1). Such expansive cell death is considered to be one of the major reasons for transplantation failures.^61^^,^^62^

### 2.2 Approaches to Stimulate Cell Survival in Tissue-Engineered Constructs

Numerous approaches have been developed to maintain viable 3D cultures. Some of them aim at enhancing the nutrient supplementation via the formation of microchannels, utilization of bioreactors, additional oxygen carriers, hyperbaric oxygen, cocultures of endothelial cells, whereas others stimulate cell growth and survival with growth factor incorporation, hypoxic priming, and preconditioning.^36^ Preconditioning usually implies a soft stress (hypoxia, acidic conditions, and nutrient deprivation), which allows cells to adapt to the subsequent unfriendly environment (observed in Ref. 63). Hypoxia stabilizes HIF-1α (hypoxia-inducible factor-1α), which is responsible for cell proliferation, differentiation, migration, survival, glucose adjustment, and vascularization.^64^ Some metabolites, such as low concentrations of $H_{2}O_{2}$ and NO, can be used as preconditioning agents against the oxidative stress.^65^^,^^66^ Mechanical stimulations—pressure, compression, and exposure to sonic waves—were shown to enhance chondrogenic differentiation.^67^ By mimicking the damaged tissue environment, the acidic conditions can stimulate cell survival, migration, and vascularization.^68^ Light preconditioning has already been utilized to increase the retinal cells’ resistance to the light stress.^69^ Moreover, PBM can act as a preconditioning agent for the other stress conditions, such as inflammation or apoptosis.^24^^,^^25^^,^^70^ Due to the involved mechanisms and achieved effects discussed in details below, PBM could be another relatively new method applied for cell preconditioning in 3D systems. Falling in the optical transparency window, the PBM of red and NIR spectrum can surpass the threshold diffusion distances (Fig. 1). Cells in the depths more than 150  μm undergo the stress, and therefore can be more susceptible to the PBM.^71^^,^^72^ Applying light to the 3D scaffolds allows to trigger cell pathways and increase survivability, implant integration, etc. For instance, NIR light successfully applied to promote integration of bone grafts in periodontal areas, skull, and osteoporotic cartilages.^22^^,^^73^^,^^74^ Even more, the vascularization of model 3D hydrogel cultures and spheroids was shown, indicating the PBM ability to provide a functional interaction between implanted graft and host tissues.^23^^,^^75^^,^^76^ PBM is already used in clinical practice to resolve chronic pain and enhance wound healing (Table 1), proving the possibility of PBM devices certification for specific purposes. Therefore, it became clear that PBM might be a promising approach to enhance the viability of cells in a 3D system.

## 3 Photobiomodulation and Scaffolds: Intersection Points

### 3.1 Irradiation Parameters and Light Sources for Photobiomodulation

The biological response to PBM strongly depends on the irradiation parameters, such as the wavelength, intensity (power per unit of irradiated area), and dose (energy per unit of the irradiated area, which can be defined by the intensity multiplied by exposure time).^17^^,^^77^ The outcome of PBM depends on the wavelength chosen. For instance, wavelengths of 623, 672, 767, and 812 nm were shown to stimulate the DNA synthesis,^78^ whereas 915 nm had no effects on the proliferation of the MG63 cell line.^79^ The dependence of the PBM effectivity on the intensity or dose can be described by the Arndt–Schultz law of biphasic intensity and the dose response.^80^ Cell growth can be enhanced in the narrow range of rather small doses ($0.17\;J/{\mathrm{cm}}^{2}$), whereas higher doses usually suppress the cell metabolic activity.^81^^,^^82^

The majority of the authors note that PBM effects do not depend on the coherency of the source.^83^^,^^84^ While it is generally the case that LED devices are considered safer to use than lasers and can be less expensive,^85^^,^^86^ with the development of electronic devices/semiconductor materials, a wide variety of semiconductor lasers (LD) appeared on the market, which, like LEDs, are cheap, easy to operate, and make it possible to create matrices for irradiating large areas and miniature wearable devices.

### 3.2 Optical Properties of Tissue-Engineered Constructs and Scaffolds

**Fig. 2:**
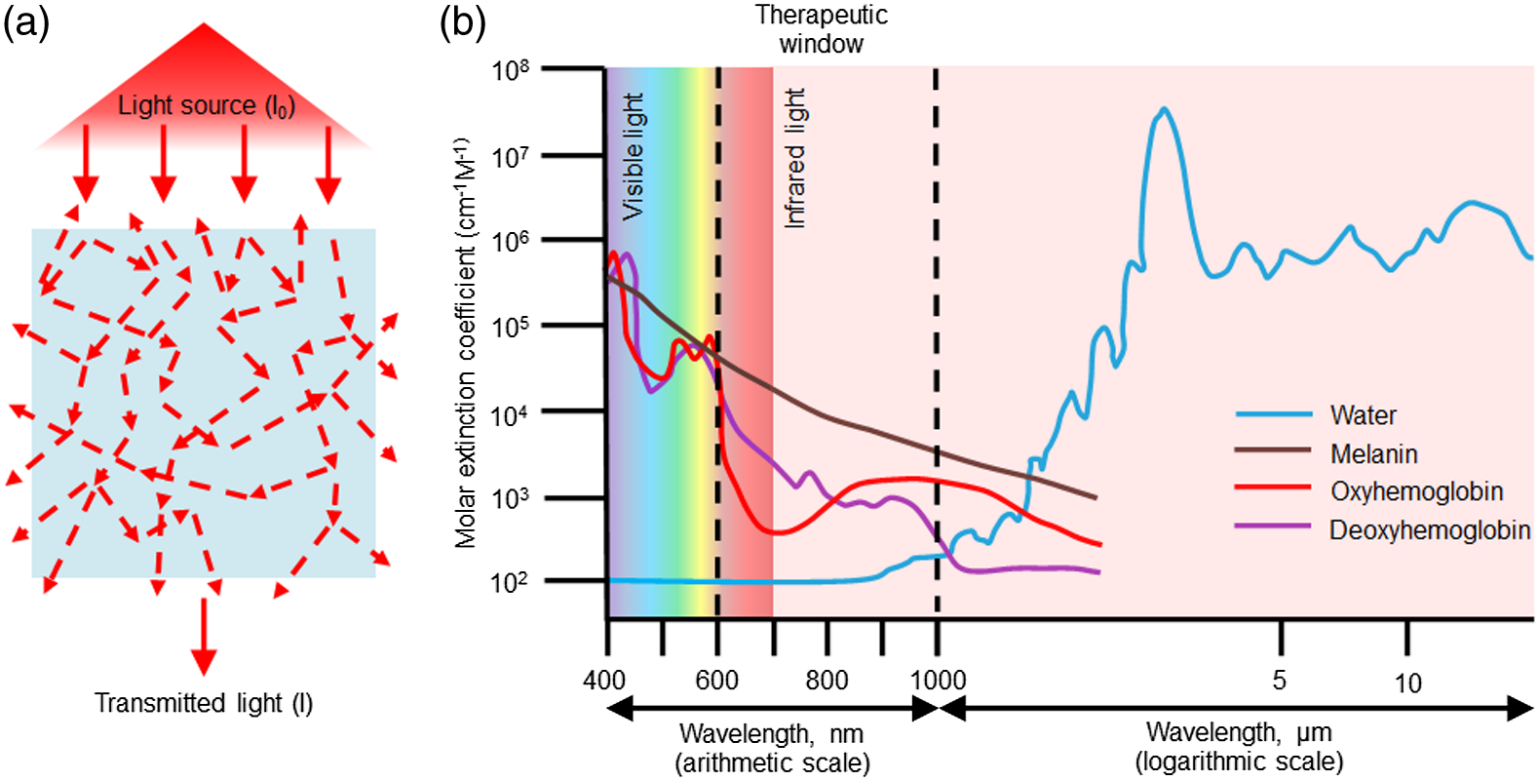
(a) the scheme of light transmittance in 3D scaffold systems. Dotted arrows indicate scattering, absorption, and reflection of light. (b) The intersection of water absorption spectrum with the PBM therapeutic wavelengths range. The optical window, where the light absorbance is minimal, is between 600 and 1300 nm. The most spread and effective wavelengths of PBM are in the range of 600 and 1000 nm.

An important feature defining the noninvasive properties of PBM is the transparency window, characterized by the penetration depths (Fig. 2). The penetration depth in tissues and scaffolds can be defined as the light path at which the intensity of the light becomes 1/e of its initial value. The light with wavelengths between 600 and 1300 nm is only slightly absorbed by water and therefore can penetrate tissues to depths up to a few centimeters.^20^^,^^21^ The average penetration of transcranial red/NIR light (630 to 810 nm) is up to 70% in mice and up to 10% in humans.^10^ The majority of scaffolds (especially hydrogels) are extensively hydrated, and consequently, they are almost transparent in visible and NIR spectral regions. However, light penetration can be significantly impacted by the tissue absorption and scattering, with the degree of reduction depending on wavelength used.^87^ Following the tissue architecture and light source parameters, light penetration can be restricted to 10 to 50 mm.^88^^,^^89^

Therefore, the exposure level for cells located in a 3D scaffold will be altered due to the light absorption and scattering in the scaffold volume. On average, the light intensity or power density is reduced with the scaffold depth. A strong difference may result in a partial exposure of the cells beyond the “therapeutic range” of PBM. To evaluate this effect, it is important to know the light intensity distribution throughout the entire scaffold’s volume.^90^

The ability of a medium to absorb and scatter photons can be described using an absorption coefficient $\mu_{a}$ and a scatter coefficient $\mu_{s}$. These coefficients are defined by the probability that the photon will be absorbed or scattered along the infinitesimal path section ds. The mean-free path for an absorption event is $1/\mu_{a}$, and the mean-free path for a scattering event is $1/\mu_{s}$.^91^ The intensity of the initially collimated beam of light (a thin beam where photons propagate in parallel) is considered to exponentially decrease with the increasing sample depth depending on the Beer–Lambert’s law:

$$I(z)=(1-R)\cdot I_{0}\cdot \exp(-\mu_{t}z),$$ (1)

where $R={\left[(n-1)/(n+1)\right]}^{2}$ is the reflection coefficient from the sample surface, n is the relative average refractive index of the sample and the environment, $I_{0}$ is the incident light intensity, $\mu_{t}=\mu_{a}+\mu_{s}$ is the total attenuation coefficient, and z is the depth.

Equation (1) represents a single-scattering approximation and is correct when $\mu_{a}\gg \mu_{s}$. Nevertheless, in tissues and scaffolds, the opposite relationship is observed: $\mu_{s}\gg \mu_{a}$ since scattering significantly predominates over absorption in the visible and NIR spectral regions. In that case, the intensity of a wide laser beam of the incident intensity $I_{0}$ at depths $z>l_{d}=1/\mu_{\mathrm{eff}}$ in a thick tissue may be described as

$$I(z)\approx (1-R)\cdot I_{0}\cdot b_{s}\cdot \exp(-\mu_{\mathrm{eff}}z),$$ (2)

where $\mu_{\mathrm{eff}}={\left[3\mu_{a}({\mu_{s}}^{\prime }+\mu_{a})\right]}^{1/2}$ is the effective attenuation coefficient, ${\mu_{s}}^{\prime }=(1-g)\mu_{s}$ is the reduced (transport) scattering coefficient, g is the scattering anisotropy factor (mean cosine of the scattering angle), and $b_{s}$ accounts for the additional irradiation of the upper layers of a tissue due to backscattering (photon recycling effect).^92^

In real cases, wide laser beams are used for PBM of highly scattering tissues with low absorption. As a result, continuous light energy is accumulated in the tissue due to the high multiplicity of chaotic long-path photon migrations. The light intensity within the superficial zone of the tissue may substantially (up to five times) exceed the incident intensity $I_{0}$.^93^ The cells, therefore, are exposed to various doses of PBM depending on their position inside the scaffold. It should also be noted that the intensity distribution within a tissue or scaffold depends not only on the sample’s optical properties and the light wavelength but also on the illumination geometry.^94^

However, in the actual case of a biological tissue or scaffold, the light scattering coefficient significantly exceeds the absorption coefficient, and the Beer–Lambert law could not be applied correctly. In this case, a more relevant mathematical description is the diffusion approximation to the radiative transfer equation. ^95^ The diffusion theory provides a good approximation for small scattering anisotropy factor g≤0.1, whereas for many tissues g≈0.6 to 0.9 and can be as large as 0.990 to 0.999 for blood. It should also be noted that the diffusion approximation does not allow one to describe boundary effects. This significantly restricts the applicability of the diffusion approximation.^95^

For modeling photon migration in turbid media, especially in bio-optical imaging applications, the Monte Carlo calculation method can be effective.^95^^,^^96^ Random migrations of photons inside a sample can be traced from their input until absorption or output occur. Using the given initial and boundary conditions, as well as the known optical characteristics of the material, this method makes it possible to calculate the distributions of light intensity and absorbed energy in samples of polylactide scaffolds and tissues.^90^^,^^95^^,^^97^ Unfortunately, Monte Carlo-based photon migration is significantly limited by the low computational efficiency.

With the growing incident beam diameter, $I_{0}$ (initial intensity) increases, leading to higher $I_{z}$ (intensity inside the scaffold). Thereby, cells in the volume of the scaffold are irradiated more evenly. Accordingly, light sources with larger apertures are more suitable for medical purposes. Lasers emit a narrow-band monochromatic light with full width at half maximum (FWHM)≪1  nm. In that case, a system of lenses or telescopic beam expanders are required for the light beam expansion.^98^^,^^99^ An LED (nonmonochromatic light with FWHM often in the range of 20 to 50 nm) or LD (monochromatic light with FWHM<3  nm) are an alternative here. These semiconductor sources usually have a small area ($<1\;{\mathrm{mm}}^{2}$) and LED/LD matrices or integrated optical components may be used to shape its radiation pattern.^100^^–^^102^ LED and LD matrices are also more efficient for homogeneous irradiation of large areas, but, without special shapers, light intensity from laser devices often has a Gaussian shape with a maximum irradiance at the center and decreased irradiance on the periphery.

It is known that the efficiency of PBM, in addition to other parameters, significantly depends on the wavelength of light.^103^^,^^104^ In the case of a biological tissue or scaffold, this relation is also superimposed on the wavelength dependence of the distribution of intensity and absorbed energy inside the medium. Therefore, in a real case, the efficiency with which light causes biochemical changes in the volume of biological tissue will significantly depend not only on the illumination (light intensity) on the “input” surface of the object but also on the selected wavelength.

Thus, precisely controlling the PBM parameters is crucial to predict the cell behavior, especially in the presence of a 3D scaffold. Although the majority of hydrogels are optically transparent to red and NIR wavelengths, light scattering can disturb the uniformity of the PBM exposure. In such cases, LED or LD sources are favorable, representing a simple, flexible, and reproducible system for the cell physiology stimulation.

## 4 Cellular Mechanisms of Photobiomodulation

The PBM mechanisms have been investigated for a long time; however, there is still no clear understanding on all the PBM pathways. The reason for that lies in a high variability of applied PBM parameters, biological objects used, and cell environment influencing on light delivery. In general, a long list of PBM targets exists, including cell surface channels and receptors, mitochondrial chromophores, and extracellular enzymes reviewed in Refs. 18 and 72. Targets, such as transient receptor potential channels, cryptochromes, and opsins, are usually react with light in green and blue spectrum. Antioxidant enzymes, namely glutathione, superoxide dismutase, and catalase, are often present in the tissue extracellular space and can be activated or inhibited in response to PBM to reduce inflammation.^105^^–^^107^ Here, we focus on the red and NIR PBM mechanisms connected to the mitochondrial chromophores since this pathway represents the most interesting for cells in 3D structures.

### 4.1 Primary Acceptors of Red and Near Infra-Red Light in a Cell

CCO in the mitochondrial electron transport chain is considered the most essential acceptor of red and NIR light in cells.^18^^,^^108^ This complex is responsible for the electron transfer from cytochrome c to molecular oxygen and can modulate redox processes in the cell.^18^ CCO, or complex IV, contains light-absorbing heme and copper centers.^109^^–^^111^

### 4.2 Secondary Messengers Activated by Light

The initial biochemical processes initiated by red or NIR light relate to CCO itself. In the conditions of the oxidative stress or inflammation, iNOS (inducible NOS, type II NOS) is assembled to produce nitric oxide (NO).^112^ NO acts as an antioxidant, controlling free radical levels in the lipid peroxidation processes, relaxes blood vessels’ walls, regulates enzymes, induces endothelial cell differentiation and modulates inflammation.^109^^,^^113^ NO can bind to CCO and reversibly inhibit it, which results in reduced mitochondrial respiration.^114^^,^^115^ PBM can induce photolysis of the CCO–NO complex, leading to the CCO release and stimulation of the electron transport chain activity^116^ followed by the mitochondrial membrane potential increase, which facilitates production of ATP, ROS, and accumulation of ${\mathrm{Ca}}^{2+}$.^108^ Moreover, photoproduced NO can take part in the regulation of the cellular pathways.

After the PBM-induced and CCO-mediated stimulation of the mitochondrial electron transport chain, mitochondria can convert more oxygen molecules ($O_{2}$) to reactive oxygen species (ROS), such as a superoxide radical ($O_{2^{-}}$).^117^^–^^119^ High concentrations of ROS are harmful to cells, however, small amounts can regulate the cell physiology.^120^

### 4.3 Cellular Pathways Triggered by Photobiomodulation

**Fig. 3:**
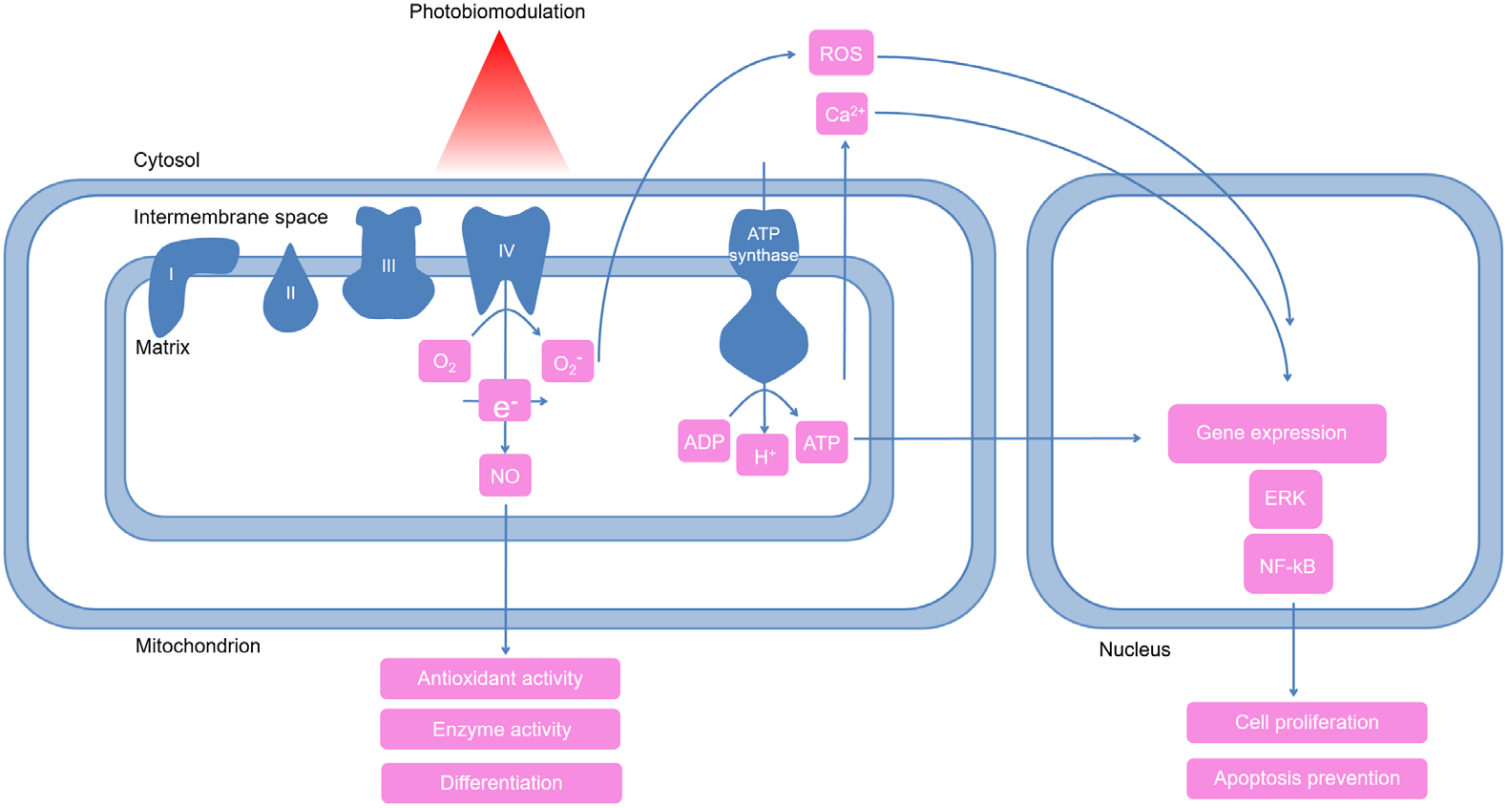
Primary events in cells induced by red and NIR light. First, light is absorbed by CCO (unit IV) and as a result, an inhibitory molecule of NO is released.^111^ NO can promote endothelial cell differentiation and regulate various enzymes. Moreover, the activity of CCO increases and the transport of electrons within the respiratory chain is stimulated. As a result, more the leakage of electrons increases and oxygen molecules ($O_{2}$) are converted into superoxide radical ($O_{2^{-}}$). Reactive oxygen species can facilitate release of mitochondrial ${\mathrm{Ca}}^{2+}$ into cytosol.^108^ All of these factors, NO, reactive oxygen species, ATP, and ${\mathrm{Ca}}^{2+}$, can act together or individually as mediators of cellular pathways and lead to activation of proliferation, cell differentiation, protection of oxidative cell damage, and modulation of apoptosis.

One of the most pronounced metabolic effects of PBM is the increased ATP production.^117^ ATP, as a source of energy, maintains the cell metabolism by itself and is also implicated in the protein and DNA synthesis, gene expression, and stimulation of the ERK1/2 pathway. PBM often increases the concentration of intracellular ${\mathrm{Ca}}^{2+}$ due to its release from the intracellular stores.^121^^,^^122^ Intracellular calcium takes part in the cell cycle regulation, cytoskeleton changes, and activation of the cellular pathways, for instance, changes in the ${\mathrm{Ca}}^{2+}$ concentration is an important mitogenic signal.^123^ NO, ATP, ${\mathrm{Ca}}^{2+}$, and ROS, as secondary messengers, are involved in various cellular pathways, leading to a wide range of downstream effects (summarized in Fig. 3). These effects include increased proliferation (via the MAPK11 cellular pathway), resistance to the oxidative stress, antiapoptotic processes, respiratory chain regulation, and DNA repair.^124^

Many PBM-inducible pathways are related to redox processes and therefore ROS production. NF-κB (nuclear factor kappa B) is one of these; it regulates numerous physiological processes, such as apoptosis, differentiation, proinflammatory genes expression, and responses to the oxidative stress.^125^ ROS-dependent NF-κB activation triggers epigenetic mechanisms via histone acetylation.^126^ Other pathways activated by changing the redox status include protein kinases, growth factors, chemokines, and more.^127^ Activation of ROS-dependent processes is restrained by the level of antioxidants, in particular.^128^

Depending on the PBM parameters, cell type and its redox status, external conditions, and other factors, cells can respond to light in different ways. One of the most frequent effects of PBM is stimulation of proliferation. It has been shown on different cells subjected to various PBM conditions. This effect is wavelength-dependent; stimulation of proliferation was observed only for red and NIR light but not for green and blue light of the same intensity.^117^^,^^129^ Cell proliferation was driven by PI3K/PKB, PI3K/Akt, Ras/Raf/ERK, PKC, Notch-1 pathway activation or D1, E, and A cyclin expression.^130^^,^^131^ Usually, these processes are mediated by ${\mathrm{Ca}}^{2+}$ signaling.

Differentiation is an *in vitro* effect of PBM opposite to proliferation. The most often applied treatment involves a combination of PBM with classical differentiation inducers and results in earlier expression of specific markers.^132^^,^^133^ The high efficacy of PBM was shown for endothelial differentiation due to the eNOS stimulation and NO formation.^134^

Another beneficial feature of PBM is the ability to inhibit apoptosis, primarily via the modulation of the Bcl-2 and Bax protein expression.^135^^,^^136^ Besides the biochemical outcomes of PBM, it can be involved in the regulation of the mitochondrial fusion and fission balance. Fusion provides protection from the nutritional and oxidative stress, autophagy, apoptosis, and mitochondrial mutations.^137^ Excessive fission or fragmentation can lead to reducing the respiratory activity and is involved in the apoptosis initiation.^138^ PBM was shown to increase the expression of MFN2, one of the proteins responsible for mitochondrial fusion.^139^

## 5 PBM for Tissue Engineering and Regenerative Medicine

The first issue to be addressed regarding PBM applications is its variety depending on different types of cells. For instance, human bone marrow-derived mesenchymal stem cells and adipose-derived stem cells (hASCs) respond oppositely to the equal PBM exposure. In the first case, proliferation intensifies, whereas in the second case, cell viability is reduced.^140^ The composition and structure of the utilized 3D systems should also be considered when choosing the proper cell type and PBM mode. For example, one should expect the osteogenic differentiation in rigid mineral-containing scaffolds, the neural differentiation in soft hydrogel systems, and the angiogenic differentiation in fibrin-based hydrogels.^141^^–^^144^

One of interesting PBM effects, which can be of great practical importance, is its protective ability. It was reported in neurotraumas and neonatal hypoxia-ischemia.^24^^,^^145^ PBM is mentioned to involve preconditioning mechanisms similar to ischemia, hyperthermia, hypothermia, and hyperbaric oxygen and is associated with increasing ATP levels, preventing mitochondrial fragmentation and cytochrome c release.^24^^,^^25^ The PBM effect is most pronounced in weakened cells cultured under nutrient- and growth factor-deficient conditions, which are usually modeled by reducing the serum concentration in the medium.^146^^–^^149^

The morphology, physiology, and behavior of cells in scaffolds are determined by the chemical structure of the material, the local topography, architecture, and mechanics of the scaffold.^150^ All of these scaffold properties can either enhance or silence the PBM effects.^151^ Despite the overall heterogeneity of the PBM parameters reported in papers, some general principles can be revealed. Most of the research is aimed at the stimulation of cell proliferation (to reach a high cell density prior to transplantation of the tissue-engineered construct) or differentiation (to integrate the construct in the host tissue). The transition between proliferation and differentiation is a crucial moment for the cell physiology and can be regulated by PBM.^152^^,^^153^ Unfortunately, the precise combinations of cell type and particular PBM mode for the specific purpose (e.g., activating proliferation or directing differentiation) are absent now. However, it is known that PBM effects depend on cell type, namely redox systems of the cell.^72^^,^^154^^–^^156^ A recent study has shown the different dynamics and effects of the same PBM mode applied to normal fibroblast or cancer cell lines.^157^ The presence of varying metabolic pathways, such as prevailing glycolysis in cancer cell lines, drastically changes the final PBM outcome. Rupel et al. showed that the redox state of the cell can determine the levels of ROS production in cells in response to PBM.^103^ Altered mitochondrial state, e.g., caused by exposure to specific mitochondrial complex inhibitors, leads to the various responses. Different inhibitor concentrations either stimulate mitochondrial activity or decrease it even more depending on inhibitor concentration.^71^ Moreover, even the amount of mitochondria in cell was connected to observed variabilities in PBM effects.^158^ Therefore, the careful choice of cell source, cell type, culturing conditions, and PBM parameters are crucial to predict the cell behavior following PBM.

### 5.1 Light-Induced Cellular Events Providing the Conditions for Effective Tissue Engineering

#### 5.1.1 Cellular proliferation

Cell proliferation is vital for tissue-engineered constructs to achieve viable structures. PBM was shown to maintain the MSCs cell cycle after implementation on a BMP-incorporated scaffold up to the sixth day of cultivation, which resulted in expanded mineral deposition.^159^ Similar results were reported in Ref. 160, where ADSCs seeded on an acellular dermal matrix were shown to proliferate and osseointegrate better after the exposure to 633 nm PBM. NIR PBM is also able to influence fibroblast proliferation through the activation of EGF expression.^161^ Both red and NIR PBM stimulate metabolic activity and proliferation of gingival MSC encapsulated in a fibrin hydrogel.^101^ It seems that PBM activates cell division in sufficiently soft scaffold systems, such as decellularized dermal matrices and hydrogels.^160^^–^^162^

#### 5.1.2 Enhanced cellular differentiation

Numerous studies are dedicated to the stimulation of bone regeneration using PBM, including the exposure of a damaged area without cells transplanted on a scaffold. The aim of PBM, in that case, is to stimulate host cells in bone defects and surrounding tissues. NIR light (730 to 830 nm) increases the efficacy of a titanium scaffold osseointegration in an osteoporosis model, skull bone reparation, and the engraftment of an autologous bone construct.^22^^,^^74^^,^^163^ PBM also helps to organize the surrounding connective tissues in the area of a bone matrix-fibrin construct implantation.^164^ Increased levels of transforming growth factor-beta (TGF-b), fibroblast growth factor-2 (FGF-2), osteoprotegerin (OPG), receptor activator of nuclear factor κB (RANK), osteocalcin (OCN), and BMP-9 in injured bone tissues after PBM were reported.^165^^,^^166^ In addition, PBM with the wavelength of 780 nm for a ceramic bone graft increased deposition of calcium hydroxyapatite and decrease of the organic components, which is important for healing of fractured bones.^167^

Similar results were observed for cell-loaded scaffolds. When irradiated with red PBM, MSCs differentiate in the osteogenic direction faster in the case of coralline biomatrices, PLGA scaffolds, and an agarose gel.^168^^–^^170^

Similarly to the findings discussed above, PBM with the wavelength of 780 nm accelerated the integration of a demineralized bone matrix graft in the periodontal area after alveolar reconstruction surgeries.^73^ Some works show that PBM increases the expression of odontogenic markers, such as DSPP, Osterix, RUNX2, BMP-2.^153^

Unlike for osteogenic and odontogenic cells, for neuronal cells, the most preferable 3D system is a hydrogel. It was shown that under the exposure to NIR PBM, embryonic neurons on a hyaluronic acid-based gel acquire adult neuronal morphology.^171^ PBM induces the neuronal differentiation and inhibits the glial differentiation of neural stem cells cultivated in a GelMA/PEGDA gel.^172^ Red irradiation combined with cross-linked gelatin loaded with ceramic particles is promising for nerve recovery. An increased nerve fiber diameter, myelin sheath thickness, and reduced muscle atrophy around the nerve was noted after PBM.^173^

A few works showed promoted vascularization of HUVECs and human ASCs cocultures in fibrin gels after red PBM.^23^ On the other hand, there are some data indicating that PBM has no influence on the angiogenic differentiation of endothelial cells.^174^ Both angiogenesis and dentinogenesis of the dentin-pulp complex were shown in a human tooth slice-based in vitro model for 810 or 660 nm light with a $1\;J/{\mathrm{cm}}^{2}$ intensity.^175^

#### 5.1.3 Anti-inflammatory effects

PBM effects on the inflammation processes were broadly studied in different animal models, e.g., burn injury, acute lung injury, and lung fibrosis. The levels of proinflammatory factors, such as TNF-α, NF-kB, IL-6, IL-1β, decreased after red or NIR irradiation.^176^^–^^179^ It also has been shown that preconditioning with PBM results in reduced levels of proinflammatory cytokines after the induction of inflammation with LPS.^70^ The anti-inflammatory activity was revealed also for immune cells. For instance, in the model of lung fibrosis, PBM therapy resulted in reduced infiltration of immune cells into alveolar capillaries.^179^ NIR PBM was able to switch M1 (inflammatory) to M2 (anti-inflammatory) polarization of macrophages.^180^ Similar effects were shown for PBM *in vitro*: red irradiation induced the transcription of IL-1β and IL-6 mRNA and decreased that of IL-8 in a cultured analog of human skin.^181^

Although the data considering inflammation processes in connection with scaffolds are limited, there is a reason to believe in the effectiveness of PBM. First of all, PBM would be useful to reduce inflammation during transplantation.

#### 5.1.4 Biopolymers organization

Since PBM can affect various signaling pathways, biopolymers undergo restructuring, subsequently altering cell behavior. Being both a dynamic structure and a crucial participant of cell signaling pathways, the cytoskeleton is the primary system of biopolymers responding to PBM. Red PBM was shown to induce the arrangement of F-actin molecules.^133^ Perhaps, this mechanism underlies the PBM-induced migration of cells within hydrogels.^182^ Similar effects were shown in respect to the ECM production. PBM leads not only to the collagen expression and synthesis^161^^,^^182^ but also to more organized aggregates in comparison to nonirradiated cells.^22^ The PBM effects in various conditions are summarized in Table 2.

**Table 2:** Parameters and effects of PBM in scaffolds.

Cell type	Scaffold	Source type	Wavelength (nm)	Energy density (fluence) ($J/{\mathrm{cm}}^{2}$)	Power density ($\mathrm{mW}/{\mathrm{cm}}^{2}$)	Cultivation after treatment	Result	Ref.
DPSC	Pluronic^®^ F-127 hydrogel incorporated with BMP4	Laser	660	3 or 5	710	21 days in culture 8 weeks in animals	Increased cell proliferation, acceleration of odonto/osteogenic differentiation	159
Primary gingival fibroblasts	Collagen matrix	LED	780	0.5; 1.5; 3	25	6 days in culture	Enhanced gene expression of hCOL-I and hEGF and increased cell viability	161
Vero (epithelial cell line)	Porcine serous collagen	LED	630	30	24.1	7 days in culture	Increased ECM deposition and proliferation	162
BM-MSC	Bio-Oss (deproteinized bovine bone)	Laser	810	4	200	3 weeks in animals	New bone formation	74
MSC	Coralline biomatrices	Laser	633	—	0.5 mW	1 to 7, 10, 14, 21, 28 days, culture	Osteogenic differentiation higher ossification levels	168
ADSC	Acellular dermal matrix	Laser	633	1	—	14, 28, 56 days in animals	Increased viability and proliferation, bone regeneration	160
NSC	GelMA/PEGDA	LED	635	—	10.95	14 days culture	Neuronal differentiation and suppressed glial differentiation	172
Embryonic NSC	NVR‐gel (cross‐linked hyaluronic acid enriched with laminin, BDNF and IGF-1)	Laser	780	—	20 to 500	24 days in culture	Increased neuronal sprouting	171
DPSC	Mg-based, Zn-doped bioceramic scaffolds	Laser	660	2 or 4, every 3 days	—	28 days culture	Increase of odontogenesis-related markers, newly formed Ca-P tissue was formed	153
HUVEC and ADSC coculture	Fibrin gel	LED	632	24	12	7 days in culture	Promoted vascularization	23
L929 and NIH3T3	Fucoidan/alginate-polyethylene glycol-gellan gum 27 (Fu/AL-PEG@GGH) hydrogel	Laser	635	4	417	1, 2, or 3 days culture	Increased cell migration, cell viability, collagen deposition	182
BM-MSC	Silk scaffolds	Polychromatic plasma arc lamp	590, 633, 666, 712, 812, 1018, 1128, 1356, 1395	—	130	7, 14, or 28 days	Osteogenic differentiation, mineral deposition, F-actin reorganization	133
ADSC	Poly-lactic-co-glycolic acid (PLGA) scaffold	Laser	660	13.3	24,62	16 weeks in animals	New bone formation, osteogenic differentiation	^169^
MC3T3 (osteoblastic cell line)	Glass-ceramic scaffold (biosilicate)	Laser	830	10	—	7 days in culture	Increased proliferation	^183^
BM-MSC	Type I collagen scaffold	Laser	810	4; every other day	430	3 weeks in animals	Bone formation	^184^
DPSC	Agarose gel	Laser	660	3.3; every 6 h	—	7 and 14 days, culture	More effective osteogenic, chondrogenic, or adipogenic differentiation	^170^

ADSC, adipose-derived stem cells; BM-MSC, bone marrow MSC; DPSC, dental pulp stem cells; MSC, mesenchymal stromal cells; NSC, neural stem cells; HUVEC, human umbilical vein endothelial cells

## 6 Perspectives

The main goal of TE is to create not only tissue-like but also fully functional structures. Although a wide range of 3D cell systems have been presented, they still lack the key features of the target tissue. The next step here is to arrange the complex architecture and cell-specific physiology. Systems such as cellular spheroids or cell sheets offer a list of advantages, including cell interactions, mechanical properties, cell phenotype preservation, etc.^185^^–^^188^ PBM could be beneficial in that case too: this approach has already been applied to spheroids, cell sheets, and organoids. 660-nm PBM induced a complex response in hASC spheroids, including HIF-1α upregulation, growth factor secretion, cytokeratin expression, angiogenesis, and vascularization in the ischemia model.^75^^,^^76^ Irradiated DPSC cell sheets expressed high levels of fibronectin and had epithelium-like cell phenotypes.^189^ These sheets also exhibited increased osteogenic differentiation.^190^ Moreover, the PBM ability to induce differentiation allowed triggering a specific direction of embryonic stem cell differentiation, which resulted in the successful formation of otic organoids.^191^ Although there is a restricted amount of such works, they are important in the context of the TE. Taking into account the beneficial effects often observed for cells in various 3D scaffolds reviewed here, the future research should be focused on the PBM utilization for the cell survival, proliferation, and differentiation in 3D scaffolds. As PBM devices are highly available, easy-to-use, tunable, and have been already certified for the clinical practice, the new combinations of biofabrication approaches with PBM are to be expected.

**Fig. 4:**
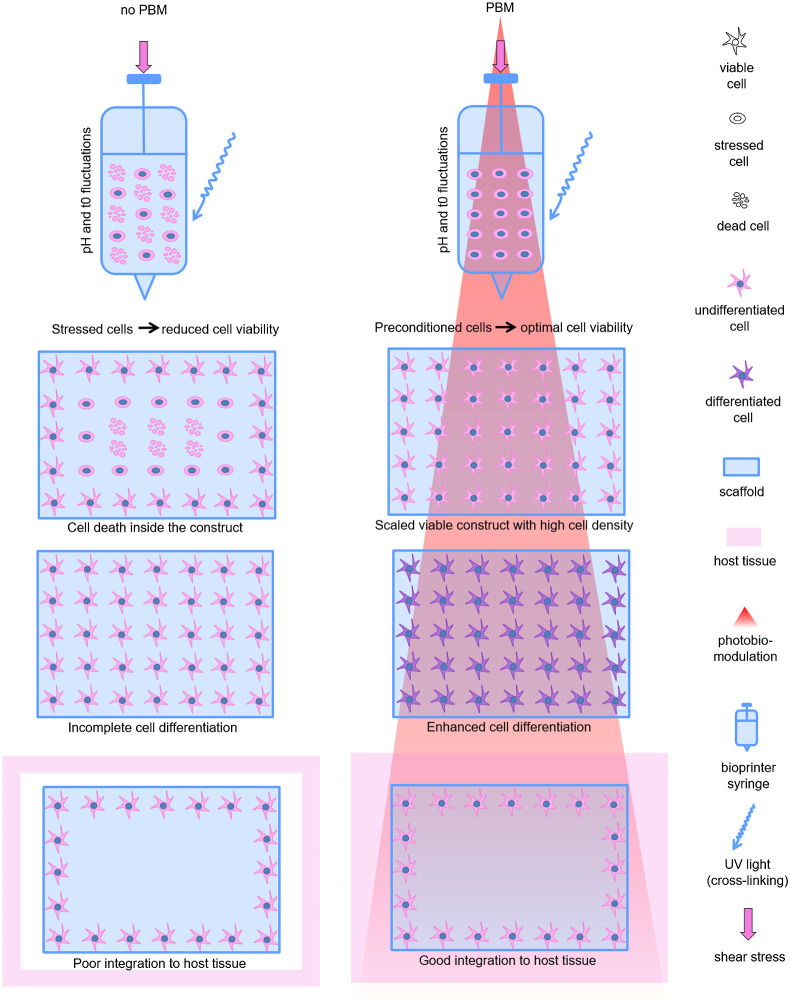
The possibilities of PBM to stimulate various aspects of cell physiology in 3D tissue engineered constructs (scaffolds or hydrogels) during the bioprinting, cultivation, differentiation, and transplantation.

However, it is still hard to predict the effects of PBM, especially in 3D systems, due to the different cell type and redox status, 3D scaffold composition, and optical properties. The PBM mechanisms involving light scattering and absorption should be clarified. However, despite all the variables, the current review suggests the high PBM potential in the field of TE, in particular for scaffolds and 3D bioprinting. Such techniques are favorable to resemble native tissue structure, but they face a few crucial limitations. Bioprinted cells suffer from shear stress, UV light during crosslinking, and temperature changes (Fig. 4). PBM sources are technically easy to introduce to bioprinters to deliver light to weakened cells in optically transparent hydrogels or scaffolds. Such modification of bioprinting approaches could increase the survivability of the scalable tissue equivalent.

PBM has currently begun being applied in numerous fields of TE, and some researchers even propose PBM as a novel fourth component of the TE triad among stem cells, scaffolds, and growth factors.^192^

## 7 Conclusion

Taken all together, scaffold-based tissue-engineered constructs and PBM complement each other. PBM stimulating wavelengths match with the optical transparency of a scaffold, and decreased cell viability after seeding in the scaffold is an object for the PBM preconditioning effect. PBM in the red and NIR ranges was shown to be effective for the stimulation of cell survival, proliferation, and differentiation in the conditions of various 3D systems. The careful selection of the PBM wavelength and intensity, coupled with the latest TE approaches, will lead to taking one step closer to creating functional and scaled tissue-like constructs.
